# Class 1 integrons and plasmid-mediated multiple resistance genes of the *Campylobacter* species from pediatric patient of a university hospital in Taiwan

**DOI:** 10.1186/s13099-017-0199-4

**Published:** 2017-09-09

**Authors:** Yi-Chih Chang, Ni Tien, Jai-Sing Yang, Chi-Cheng Lu, Fuu-Jen Tsai, Tsurng-Juhn Huang, I-Kuan Wang

**Affiliations:** 1 Department of Medical Laboratory Science and Biotechnology, China Medical University, 91 Hsueh-Shih Road, Taichung, 40402 Taiwan; 2 Department of Laboratory Medicine, China Medical University Hospital, 2 Yu-Der Road, Taichung, 40447 Taiwan; 3 Department of Medical Research, China Medical University Hospital, China Medical University, 2 Yu-Der Road, Taichung, 40447 Taiwan; 4 Department of Pharmacy, Buddhist Tzu Chi General Hospital, 707, Sec. 3, Chung-Yang Road, Hualien, 97002 Taiwan; 5 Human Genetic Center, China Medical University Hospital, 2 Yu-Der Road, Taichung, 40447 Taiwan; 6 School of Chinese Medicine, China Medical University, 91 Hsueh-Shih Road, Taichung, 40402 Taiwan; 7 School of Medicine, China Medical University, 91 Hsueh-Shih Road, Taichung, 40402 Taiwan; 8 Division of Nephrology, China Medical University Hospital, 2 Yu-Der Road, Taichung, 40447 Taiwan

**Keywords:** *Campylobacter* species, Pediatric patient, Integrons, Antibiotic resistance gene

## Abstract

**Background:**

The *Campylobacter* species usually causes infection between humans and livestock interaction via livestock breeding. The studies of the *Campylobacter* species thus far in all clinical isolates were to show the many kinds of antibiotic phenomenon that were produced. Their integrons cause the induction of antibiotic resistance between bacterial species in the *Campylobacter* species.

**Results:**

The bacterial strains from the diarrhea of pediatric patient which isolated by China Medical University Hospital storage bank. These isolates were identified by MALDI-TOF mass spectrometry. The anti-microbial susceptibility test showed that Campylobacter species resistant to cefepime, streptomycin, tobramycin and trimethoprim/sulfamethoxazole (all *C. jejuni* and *C. coli* isolates), ampicillin (89% of *C. jejuni*; 75% of *C. coli*), cefotaxime (78% of *C. jejuni*; 100% of *C. coli*), nalidixic acid (78% of *C. jejuni*; 100% of *C. coli*), tetracycline (89% of *C. jejuni*; 25% *C. coli*), ciprofloxacin (67% of *C. jejuni*; 50% *C. coli*), kanamycin (33% of *C. jejuni*; 75% *C. coli*) and the *C. fetus* isolate resisted to ampicillin, cefotaxime, nalidixic acid, tetracycline, ciprofloxacin, kanamycin by disc-diffusion method. The effect for ciprofloxacin and tetracycline of the *Campylobacter* species was tested using an E-test. The *tet, erm*, and *integron* genes were detected by PCR assay. According to the sequencing analysis (type I: *dfr12*-*gcuF*-*aadA2* genes and type II: *dfrA7* gene), the cassette type was identified. The most common gene cassette type (type I: 9 *C. jejuni* and 2 *C. coli* isolates; type II: 1 *C. coli* isolates) was found in 12 class I integrase-positive isolates.

**Conclusions:**

Our results suggested an important information in the latency of *Campylobacter* species with resistance genes, and irrational antimicrobial use should be concerned.

**Electronic supplementary material:**

The online version of this article (doi:10.1186/s13099-017-0199-4) contains supplementary material, which is available to authorized users.

## Background

The *Campylobacter* species, a *bacillus*, causes diseases in animals and humans [1]. Most species of animal including cattle, chicken, turkey, dog, cat, mink, ferret, pig, and primate is susceptible to infection [2]. Animals can be exposed to the *Campylobacter* species bacteria by direct contact with infected animals, or through contaminated feed or water. Raw or undercooked meat fed to pets may also contain the *Campylobacter* species [3]. In the 1970s, the *Campylobacter* species was first identified as a human pathogen [4, 5]. The *Campylobacter* species is a zoonotic pathogen, and it infects humans through contaminated food, water, or milk [6]. Infections caused by the *Campylobacter* species has emerged as a leading cause of acute gastroenteritis worldwide [7, 8]. The clinical characteristics of infection can create diarrhea, abdominal pain, fever, and vomiting [9]. The major pathogens of the *Campylobacter* species in humans is *Campylobacter jejuni*, *Campylobacter coli*, *Campylobacter lari*, and *Campylobacter fetus* [10]. The group with the highest incidence rate of *Campylobacter* species infection is infants younger than 2 years of age [11]. *Campylobacter* species’ infection causes moderate to severe diarrhea in infants and young children [11].

Most campylobacteriosis need not be therapy, but patient requires antibiotic treatment if the symptoms is severe. Fluoroquinolones, erythromycin, and tetracycline are the first-line antibiotic agents for treatment of *Campylobacter* species’ infections [12]. The inappropriate use of anti-microbial agents in animal husbandry has led to the development of antibiotic resistance in *Campylobacter* treatment [12]. Recently, the Centers for Disease Control and Prevention (CDC) has recorded drug-resistant *Campylobacter* as a thoughtful threat in the United States. Resistance of *Campylobacter* to antibiotics agents is interceded by multiple mechanisms [13]. The resistance mechanisms of the *Campylobacter* species bacteria include horizontal gene transfer (HGT) or multidrug efflux pump [13]. Integron is considered to be vectors for rapid HGT that causes antibiotic resistance between bacterial species [14, 15]. In Gram-negative bacteria, integrons are possibly a major factor in distribution of multidrug resistance [16]. Integron encoding the anti-microbial resistance gene (s) may act an important role for the dissemination of resistance in *Campylobacter* isolates. In addition, the class 1 integrons associated with the *aadA9* gene (aminoglycoside-resistance gene) located on an R-plasmid have been reported in *Campylobacter* isolates [12].

The aim of this study was to identify the major pathogens and to assess the molecular basis of resistance to antibiotic agents in *Campylobacter* species isolated from the diarrhea of pediatric patient in Taiwan.

## Methods

### Isolation and identification of bacterial strains

The 14 *Campylobacter* strains used in this study were isolated from feces sample of pediatric diarrhea patient in China Medical University Hospital storage bank from January 2014 to February 2017. The *Campylobacter* species was cultured on Campy-BAP plates (Becton–Dickinson and Company, USA) containing five anti-microbial agents (amphotericin B, polymyxin B, cephalothin, trimethoprim, and vancomycin) and 10% sheep blood overnight at 42 °C and in an air condition of 5% O_2_, 10% CO_2_, and 85% N_2_. Subsequently, the strains were all re-cultivated under microaerophilic conditions. The isolated strains were then placed in storage in skim milk medium containing 20% glycerol stock at a temperature of −80 °C [17, 18].

### MALDI-TOF mass spectrometry

The cultured bacteria were suspended in 300 μl of bi-distilled water and mixed with 900 ml of ethanol (Carl Roth GmbH). The measurement of the sample was then conducted following procedures previously described by El-Ashker et al. [19]. Measurement was performed with an Ultraflex III TOF/TOF mass spectrometer (Bruker Daltonics) equipped with a 200-Hz smartbeam 1 laser. The parameter setting was as follows: delay, 80 ns; ion source, 1 V, 25 kV; ion source, 2 V, 23.4 kV; lens voltage, 6 kV; and mass range 0–20 137 kDa. The raw spectra was analyzed by MALDI BIOTYPER 2.0 software (Bruker Daltonics, Billerica, MA, USA) using the default settings. The procedure of the MALDI-TOF mass spectrometry measurement was performed automatically without any user intervention. The software generated a list of peaks up to 100. The peaks with a mass-to-charge ratio difference of <250 ppm were considered to be identical. The peak list that was generated was used for matches against the reference library by directly using the integrated pattern-matching algorithms of the software. All parameters were the same regardless of the presumptive bacterial species analyzed. The BIOTYPER 2.0 database was composed using only *Campylobacter* and related species [20].

### Antibiotics resistance screening in *Campylobacter* species by Kirby–Bauer disc diffusion test and Epsilometer test

The Kirby–Bauer disc diffusion test and Epsilometer test (Etest) were used to determine the anti-microbial susceptibility of the *Campylobacter* spp. isolates [21–23]. The susceptibility of each isolate to each antibiotic was determined according to the Neo-Sensitabs™ “user’s guide” for susceptibility testing and the latest guidelines of the Clinical and Laboratory Standards Institute (CLSI). To conduct the Kirby–Bauer disc diffusion test, sixteen antibiotic discs were chosen, including amikacin (30 μg/disc), ampicillin (30 μg/disc), cefepime (30 μg/disc), cefotaxime (30 μg/disc), chloramphenicol (30 μg/disc), ciprofloxacin (5 μg/disc), erythromycin (15 μg/disc), gentamicin (10 μg/disc), imipenem (10 μg/disc), kanamycin (5 μg/disc), nalidixic acid (30 μg/disc), streptomycin (30 μg/disc), sulfamethoxazole/trimethoprim (1.25/23.75 μg/disc), tetracycline (5 μg/disc) and tobramycin (10 μg/disc). All of which were purchased from Mast Group Ltd. (Merseyside, UK). The Campy-BAP plates were prepared and inoculated with the *Campylobacter* spp. isolates according to the procedures recommended by the NCCLS. The plates were incubated at 37 °C under the same microaerophilic conditions already described in the “Isolation and identification of bacterial strains” section above for 48 h.

For the Epsilometer test (Etest), five antibiotic Epsilometer strips were used, including strips containing amikacin, ciprofloxacin, imipenem, piperacillin/tazobactam, and tetracycline. For each tested organism, a Campy-BAP plate was inoculated by swabbing the plate evenly in three directions with a 1.0 McFarland standard of the test organism. The Etest strips were then applied to the surface of the plate and were incubated under the same conditions as indicated above for the disc diffusion test [22].

### Detection of tetracycline- and erythromycin-resistant genes

The plasmid DNA was extracted using a Plasmid Miniprep Purification kit (GeneMark, GMbiolab, Taichung, Taiwan) and the genomic DNA extract was prepared with the PureLink Genomic DNA Mini kit (Carlsbad, CA, USA). The polymerase chain reaction (PCR) assay was applied to detect tetracycline-resistant genes reported by Abdi-Hachesoo et al. [24, 25]. The 9 pairs of primers were used to detect *tet (A), tet (B), tet (K), tet (L), tet (M), tet (O), tet (Q), tet (S),* and *tet (W)* gene in plasmid and genomic DNA of *Campylobacter* isolates, respectively. The specific genes, applicons, primers, and annealing temperatures are listed in Additional file 1: Table S1. Briefly, we performed the PCR with O’In 1 DNA polymerase (Yeastern Biotech, Taipei, Taiwan) with 10 pmol of forward and reverse primers (Mission Biotech Co., Ltd., Taipei, Taiwan) and 60 ng of the extracted plasmid or chromosomal DNA. The *Campylobacter jejunii* isolate 3 was positive for *tet (A), tet (L), tet (M), tet (O),* and *tet (Q)*, we further identified the PCR product by DNA sequencing. We used the *Campylobacter jejunii*-isolate 3 as positive control for these genes. The erythromycin-resistant (*erm*) genes were detected, and the PCR-amplification of primers for *erm (A), erm (B), erm (C),* and *erm (F)*, respectively, were used to detect any *Erm*-*R* genes in the individual *Campylobacter* isolates. Each gene of the PCR conditions, primers, and size that was listed in Additional file 1: Table S1.

### The class 1 integrase and integron detection

The gene cassettes of integron were harbored in all the integrase-positive isolates. In order to screen these isolates for the presence of the class 1 integrase gene, the specific primers IntI1F and IntI1R were used [26]. The specific oligonucleotide PCR primer used in this study is listed in Additional file 1: Table S1. Following the manufacturer’s instructions, template DNA from all the isolates was prepared using Plasmid Miniprep Purification kits (GeneMark). PCR was then performed in a total volume of 25 μl consisting of 1 μl of target DNA, 17 μl of distilled water, 1 μl of each primer (10 μM), and 5 μl of 5× Master Mix (PCR Master Mix Kit, GeneMark), which in turn consisted of 0.75 U Taq Plus DNA polymerase, 250 μM dNTP, 2 mM MgCl_2_, and PCR buffer. More specifically, the amplicons that were used for the gene cassette of class I integron analysis were determined by the specific primers Cassette F and Cassette R [27], while the specific oligonucleotide PCR primer used is listed in Additional file 1: Table S1. The PCR procedures were executed under the same conditions as indicated above for the integrase gene detection (except that the annealing temperature was set at 55 °C for 30 s for detecting the *intI1* gene while it was set at 57 °C for 45 s for detecting the gene *cassette*). PCR products of integron gene cassette were then sequenced from both sides. The same company (i.e., Mission Biotech Co., Ltd.) also synthesized the oligonucleotide primers used for the DNA sequencing. The nucleotide sequences were analyzed using the Blast program available on the National Center for Biotechnology Information (NCBI) website (http://blast.ncbi.nlm.nih.gov/Blast.cgi) in order to search for the closest sequences in the GenBank database (NCBI, US National Library of Medicine).

### Molecular typing

The genotyping of the 14 *Campylobacter* species strains was performed according to a standard operating procedure which was recommended by the Centers for Disease Control and Prevention (CDC) for PulseNet PFGE of *Campylobacter* spp. (http://www.cdc.gov/pulsenet). The DNA of agarose plugs was performed with *SmaI* restriction enzyme. The electrophoresis conditions consisted of an initial switch time of 6.8 s and a final switch time of 35.4 s and a gradient of 6 V/cm for 19 h on CHEF-DR III System (Bio-Rad, Hercules, CA, USA). The PFGE patterns were analyzed using Gel Compare II version 6.5 software (Applied Maths, Sint-Matenslatem, Belgium). The DNA size marker was used by standard Salmonella serovar Branderup H9812 strain (digested with *Xba*I restriction enzyme). The pair comparisons of types and cluster analyses were performed by the Dice correlation coefficient and UPGMA (unweighted pair group method with arithmetic averages) clustering algorithm.

## Results

### The *C. jejuni*, *C. coli*, and *C. fetus* strains are the major *Campylobacter* species in the diarrhea of pediatric patient

According to the MALDI-TOF mass spectrometry results, the detection ratio of the clinical isolates for all the patient treated from January 2015 to December 2015 was 5%. The bacterial species of clinical isolates were identified by the adequate level (score ≥2.0; the addition of biochemical tests for score ≤2.0). In total samples, ten *C. jejuni* strains, three *C. coli* strains, and one *C. fetus* strain were isolated. Overall, the respective population rates for the *C. jejuni, C. coli,* and *C. fetus* strains were 71% (10/14), 21% (3/14), and 8% (1/14). These results indicate that *C. jejuni, C. coli,* and *C. fetus* strains were the major dominant *Campylobacter* species found in the samples from the diarrhea of pediatric patient.

### Anti-microbial susceptibility patterns of *Campylobacter* isolates

The Kriby–Bauer disc diffusion test was used in this experiment. More specifically, a variation of the standard disc diffusion method that has been approved by the CLSI and EUCAST as an acceptable means of screening for the susceptibility of *Campylobacter* isolates so as to be treated with fifteen antibiotics, and the result is summarized in Table 1. It should be noted that because the breakpoints for amikacin, cefepime, streptomycin, and tobramycin have not yet been defined by the CLSI, the suggested *E. coli* breakpoint was used for those particular antibiotics instead. All the *Campylobacter* isolates exhibited full resistance to cefepime, streptomycin, and trimethoprim/sulfamethoxazole, as well as potential resistance to ampicillin, ciprofloxacin, cefotaxime, kanamycin, nalidixic acid, tobramycin, and tetracycline. Conversely, all the isolates were susceptible to amikacin, chloramphenicol, erythromycin, gentamicin, and imipenem.

**Table 1:** The disc diffusion test of *Campylobacter specie* isolates from China Medical University Hospital stored bank

Antibiotics	Resistance	Breakpoints (mm)
Amikacin (AK)	4 (29%)	14–17
Ampicillin (AP)	12 (86%)	23–28
Cefepime (CFM)	14 (100%)	18–25
Cefotaxime (CTX)	12 (86%)	23–28
Chloramphenicol (C)	5 (36%)	23–28
Ciprofloxacin (CIP)	9 (64%)	20–24
Erythromycin (E)	5 (36%)	12–16
Gentamicin (GM)	3 (21%)	12–15
Imipenem (IPM)	5 (36%)	23–28
Kanamycin (K)	7 (50%)	13–18
Nalidixic acid (NA)	12 (86%)	13–19
Streptomycin (S)	14 (100%)	4–32
Tobramycin (TM)	13 (96%)	18–26
Trimethoprim/sulfamethoxazole (SXT)	14 (100%)	23–28
Tetracycline (TE)	10 (71%)	22–26

All the isolates were tested by Epsilometer test (Etest) for their susceptibility to four antibiotics (amikacin, ciprofloxacin, imipenem and tetracycline), and the result is summarized in Additional file 1: Table S2. Because the breakpoints for amikacin and imipenem have not yet been defined by the CLSI, the suggested *E. coli* breakpoint from the same reference paper was used instead. All the isolates showed potential resistance to ciprofloxacin and tetracycline, while only five isolates exhibited resistance to amikacin, and only two isolates exhibited resistance to imipenem. Our prediction is that the will be isolates susceptible to amikacin and imipenem.

### Tetracycline and erythromycin-resistant genes in plasmid

**Fig. 1:**
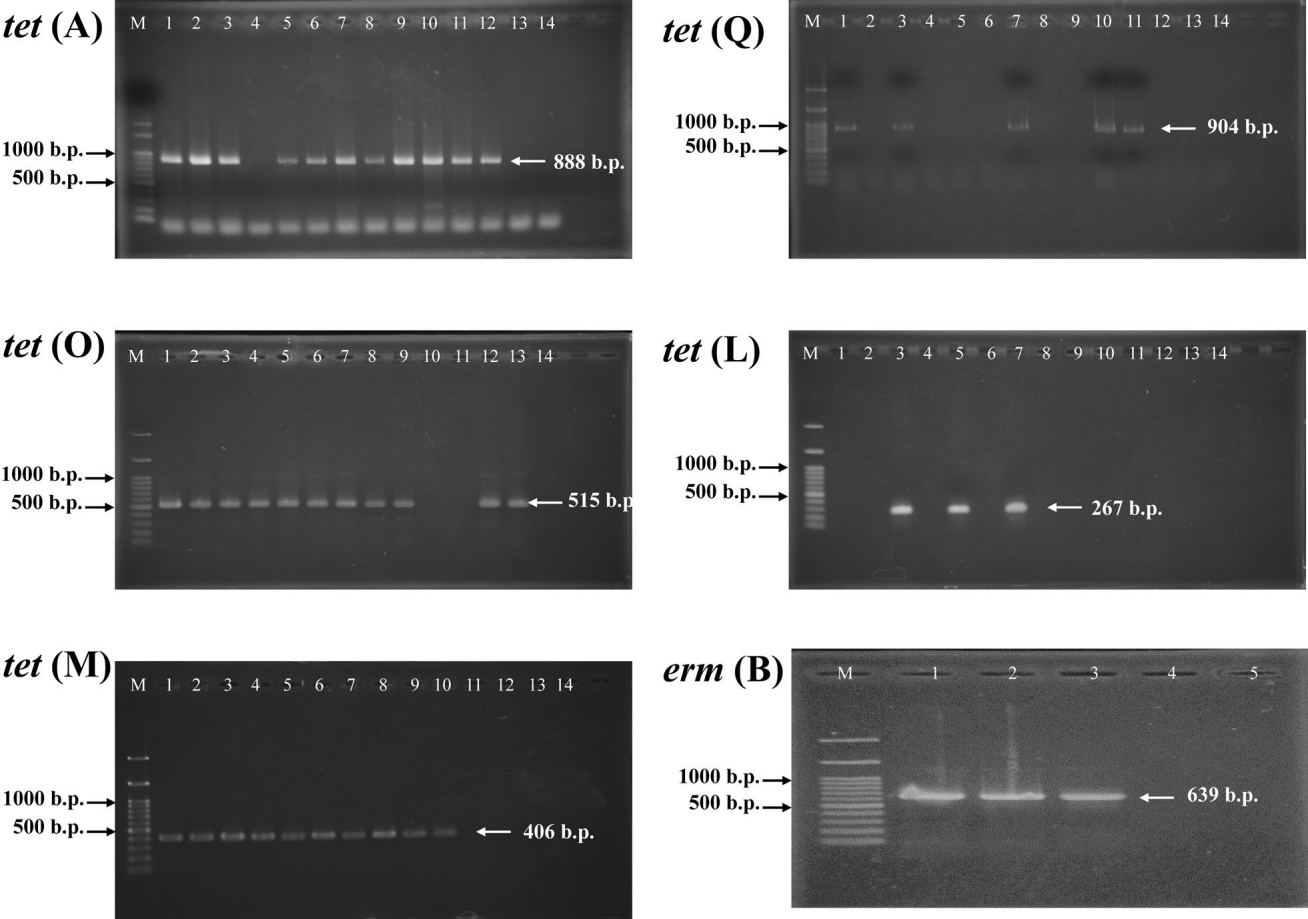
Polymerase chain reaction amplification of the *tet (A)* gene, t*et (O), tet (M), tet (Q), tet (L)* and *erm (B)* gene. (M: 100 b.p. DNA marker; *Campylobacter jejuni*: *lines 1–6*, *9*, *11*, and *12*; *Campylobacter coli*: *lines 7*, *8*, *10*, and *13*; *Campylobacter fetus*: *line 14*)

All *Tet*-*R* genes were found in plasmid, but not in genomic DNA, of tested *Campylobacter* spp. isolates. According the results of the screening for tetracycline-resistant genes, 79% (11/14) of the *Campylobacter* spp. isolates tested positive for *tet (A)* and *tet (O)* total, with *tet (A)* being seen in 89% (8/9) of the *C. jejuni* isolates and 75% (3/4) of the *C. coli* isolates. The *tet (O)* was found in 89% (8/9) of the *C. jejuni* isolates, 50% of the *C. coli* isolates, and 100% of the *C. fetus* isolates. Ten of the *Campylobacter* spp. isolates (71%) total tested positive for *tet (M)*: it was detected in 78% of *C. jejuni* isolates (7/9) and 75% of the *C. coli* isolates (3/4). The *tet (Q)* resistant genes were seen in 36% of these *Campylobacter* spp. isolates total, being found in 33% of the *C. jejuni* isolates (3/9) and 50% of the *C. coli* isolates (2/4). The *tet (L)* (Fig. 1), which had the lowest prevalence rate, was found in only three of the *Campylobacter* spp. isolates total (21%), being detected in 22% of the *C. jejuni* isolates (2/9) and 25% of *C. coli* isolates (1/4). The *Campylobacter* isolates were screened to determine which harbored any of nine tetracycline-resistant genes, and the result is summarized in Additional file 1: Table S3. The *tet (B), tet (K), tet (S)* and *tet (W)* resistant genes were not identified in any of the tested *Campylobacter* spp. isolates.

The result of the PCR detect *Erm*-*R* genes in 36% (5/14) erythromycin-resistant isolates showed that only the *erm (B)* (Fig. 1) (2 *C. jejuni* isolates, 22%; 1 *C. coli* isolate, 25%) was found (Additional file 1: Table S4). The *erm (A), erm (C),* and *erm (F)* resistant genes were not identified in any of the tested *Campylobacter* spp. isolates.

### Class 1 integron detection in *Campylobacter* species

In Additional file 1: Table S5, we show the characteristics of various gene cassette types in class 1 integron of *Campylobacter* species isolates. The *integrase* gene *(intI1)* was detected in 86% of the fourteen *Campylobacter* isolates (12/14) identified in this study. Among the *intI1*-positive strains, twelve isolates (86%) harbored class 1 integron containing various sizes of gene cassettes ranging from 750 to 1907 b.p. in length.

**Fig. 2:**
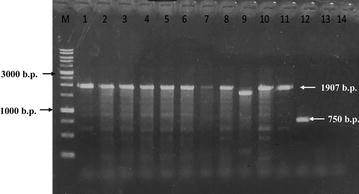
Polymerase chain reaction amplification of the class I *integron* gene cassette. (M: 1000 b.p. DNA marker; *Campylobacter jejuni*: *lines 1–6*, *9*, *11*, and *12*; *Campylobacter coli*: *lines 7*, *8*, *10*, and *13*; *Campylobacter fetus*: *line 14*)

The results of the PCR testing to detect *Class I integron cassette* genes indicated that 86% (12/14) of the *Campylobacter* spp. isolates tested positive for *Class I integron cassette* genes (Fig. 2). According to the sequencing analysis, two gene cassette types were identified in this study (namely, type I: *dfr12*-*gcuF*-*aadA2* genes and type II: *dfrA7* gene). Each cassette type had a unique inserted gene cassette pattern harboring various antibiotic-resistant genes. The most common gene cassette type (type I: *dfr12*-*gcuF*-*aadA2 genes*) was found in 92% of the 12 *intI1*-positive strains (9 *C. jejuni* strains and 3 *C. coli* strains) and was responsible for trimethoprim, streptomycin, and spectinomycin resistance in those strains. The other cassette type (type II: *dfrA7* gene) was found in only 8% of the *intI1*-positive strains (i.e., one *C. coli* strain) and was responsible for trimethoprim resistance in that strain.

### Pulsed-field gel electrophoresis of *Campylobacter* isolates

**Fig. 3:**
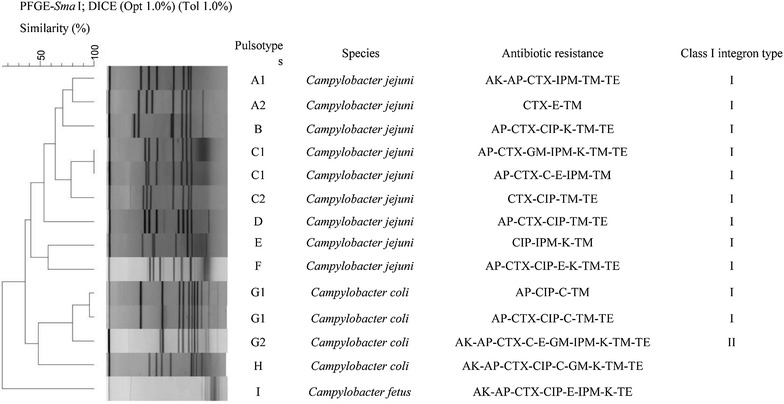
The dendrogram of *Sma*I digested PFGE profile of 14 *Campylobacter* strains. *AK* amikacin, *AP* ampicilin, *CFM* cefepime, *CTX* cefotaxime, *C* chloramphenicol, *CIP* ciprofloxacin, *E* erythromycin, *GM* gentamicin, *IPM* imipenem, *K* kanamycin, *NA* nalidixic acid, *S* streptomycin, *TM* tobramycin, *T* tetracyclin, *SXT* trimethoprim/sulfamethoxazole

In Fig. 3, among 14 *Campylobacter* isolates were typeable by PFGE, that the guidelines of PFGE patterns were applied by Tenover [28]. PFGE analysis was performed in order to determine the genetic diversity of these isolates and the relationship between the isolates retrieved from different patients. Analysis of the PFGE profiles of the *Campylobacter* isolates suggested that the isolates possessed diverse genotypes, when comparing profiles of isolates belonging to different species. The similarity of the isolates was from 50 to 100%. These 14 isolates had 13 different PFGE genotypes, only one PFGE type was recovered between two *C. jejuni* isolates. Furthermore, by using a cut-off similarity value of 75%, profiles of the *C. jejuni* were classified to 6 clusters and *C. coli* were classified to 2 clusters.

## Discussion

The *Campylobacter* species is some of the many bacterial foodborne pathogens worldwide. After *Campylobacter* infection, chronic sequelae can be very serious, causing lifelong morbidity. *Campylobacter* species infection is also associated with various chronic sequelae. In addition, *C. jejuni* in particular is among the most frequent in acute enteritis [1]. The major *Campylobacter* species in humans is *C. jejuni*, *C. coli, C. lari*, and *C. fetus* [10]. In Taiwan, Wang et al. demonstrated that in 104 enteric campylobacteriosis patient, *C. coli* was found in 24 patient (23.1%), while *C. jejuni* was found in 80 patient (76.9%) [9]. In our study, we used the MALDI-TOF–MS analysis method to determine the major *Campylobacter* species in the diarrhea of pediatric patient, with our results showing that *C. jejuni*, *C. coli,* and *C. fetus* were the major *Campylobacter* species found in our samples. Thus, our study is in agreement with previous findings.

*Campylobacter species* infection leads to campylobacteriosis, so fluid supplementation and antibiotic treatments are the most important therapies. The first-line antibiotic agent used in the treatment of *Campylobacter* infections is such as erythromycin and ciprofloxacin. Tetracycline can be an alternative choice in the treatment of clinical campylobacteriosis [29]. Antibiotic resistance in *Campylobacter* species therapy has now become a major public health concern worldwide. In this study, the disc diffusion test and the Etest were used to detect the antibiotic-resistant effect in *Campylobacter* species. Our results demonstrated that all the isolates presented resistance to cefepime, streptomycin, and trimethoprim/sulfamethoxazole, ampicillin, ciprofloxacin, cefotaxime, kanamycin, nalidixic acid, tobramycin, and tetracycline (Additional file 1: Tables S1, S2). In addition, the PCR results indicate that all the *Tet*-*R* genes and *Erm*-*R* genes were found in plasmid (Additional file 1: Tables S3, S4; Fig. 1).

Resistance to tetracycline is converted by the gene in *Campylobacter* species. The *Tet (O)* and *tet (S)* genes are transferred as plasmid-encoded genes and are not self-mobile in the chromosome. The *tet (A)* and *tet (B)*, efflux genes, code for an approximately 46-kDa membrane bound efflux protein for membrane-associated proteins that export tetracycline from the cell. Our results demonstrate that 79% (11/14) of the *Campylobacter* species isolates tested positive for *tet (A)* and *tet (O)* (Fig. 1), and *tet (A)* was seen in 80% of the *C. jejuni* isolates and 100% of the *C. coli* isolates. The *tet (O)* gene was found in 80% of the *C. jejuni* isolates, 67% of the *C. coli* isolates, and 100% of the *C. fetus* isolates. (Additional file 1: Table S3). It has been reported that the () gene was also found in 33–76% of tetracycline-resistant *C. jejuni* isolates lacking plasmids in Canada and Australia, respectively [30].

Erythromycin is the first macrolide antibiotics agent. Erythromycin inhibits bacterial RNA-dependent protein synthesis in ribosomal 50S subunit. There are two mechanisms of macrolide resistance, one is 23S rRNA and the mutations of ribosomal proteins L4 and L22, and the other is antibiotic efflux by the multidrug efflux pump CmeABC [31]. It has been reported that the rRNA methylase gene *erm* (B) mediates resistance to erythromycin in one *C. coli* isolate of farm-animal origin [31, 32]. Our results demonstrate that only the *erm* (B) was seen in *C. jejuni* and *C. coli* (Additional file 1: Table S4; Fig. 1). Thus, our study is in agreement with previous studies.

Integron is a major genetic element in multidrug-resistant Gram-negative bacteria. The integron contains an integrase gene and a site-specific integration site where the integrase can link antibiotic-resistant gene cassettes. There are nine classes of integron and over 60 distinct antibiotic-resistant gene cassettes have been characterized within integrons. Our results demonstrate that the *integrase* gene *(intI1)* was detected in 86% of the fourteen *Campylobacter* isolates (12/14). 71% (10/14) of the *Campylobacter* spp. isolates tested positive for *Class I integron gene cassette* by PCR (Fig. 2). The sequencing analysis and PCR demonstrated that two gene cassette types were identified, including type I: *dfr12*-*gcuF*-*aadA2* genes; and type II: *dfrA7* gene. It has already been reported that the class 1 integron gene is associated with antibiotic resistance in *Campylobacter jejuni* isolated from the broiler chicken house environment [33].

## Conclusions

This is the first extensive study of as well as new findings in the differential categorization of antibiotic-resistant genes and integron gene cassettes in clinical *Campylobacter* species. To our knowledge, *C. jejuni* isolate harbored *erm* (B) in plasmid was only found in strains from animal, and we first reported that patients infected with *erm* (B)-positive *C. jejuni*, two types of class I *integron* gene cassette pattern were different from previous studies. Therefore, an important information in the latence of *Campylobacter* species with antibiotic-resistant genes and the irrational using antimicrobial agents in therapy are what concerned us.

## Additional file

**Additional file 1.** Additional tables.
