# Supplementary material for: Class 1 integrons and plasmid-mediated multiple resistance genes of the Campylobacter species from pediatric patient of a university hospital in Taiwan
Source: Gut Pathog. 2017 Sep 9;9:50. doi: 10.1186/s13099-017-0199-4 (PMC5591528; doi:10.1186/s13099-017-0199-4)
Supplement: Supplementary file 1 — Additional file 1. Additional tables. [file 13099_2017_199_MOESM1_ESM.docx]

**Table S1. Primers and annealing temperatures used for detection of tetracycline, erythromycin resistance genes and *integron* gene.**

| **Gene** | **Primers** | **Gene size** | **Annealing temperature** |
| --- | --- | --- | --- |
| *tet (A)* | F : GTGAAACCCAACATACCCC  R : GAAGGCAAGCAGGATGTAG | 888 bp | 54 ^o^C |
| *tet (B)* | F : CCTTATCATGCCAGTCTTGC  R : ACTGCCGTTTTTTCGCC | 774 bp | 54 ^o^C |
| *tet (K)* | F : TCGATAGGAACAGCAGTA  R : CAGCAGATCCTACTCCTT | 169 bp | 50 ^o^C |
| *tet (L)* | F : TCGTTAGCGTGCTGTCATTC  R : GTATCCCACCAATGTAGCCG | 267 bp | 56 ^o^C |
| *tet (M)* | F : GTGGACAAAGGTACAACGAG  R : CGGTAAAGTTCGTCACACAC | 406 bp | 54 ^o^C |
| *tet (O)* | F : AACTTAGGCATTCTGGCTCAC  R : TCCCACTGTTCCATATCGTCA | 515 bp | 56 ^o^C |
| *tet (Q)* | F : TTATACTTCCTCCGGCATCG  R : ATCGGTTCGAGAATGTCCAC | 904 bp | 55 ^o^C |
| *tet* ( S ) | F : CATAGACAAGCCGTTGACC  R : ATGTTTTTGGAACGCCAGAG | 667 bp | 54 ^o^C |
| *tet* (W) | F : GAGAGCCTGCTATATGCCAGC  R : GGGCGTATCCACAATGTTAAC | 168 bp | 58 ^o^C |
| *erm* (A) | F : CTTCGATAGTTTATTAATATTAGT  R : TCTAAAAAGCATGTAAAAGAA | 645 bp | 48 ^o^C |
| *erm* (B) | F : AGTAACGGTACTTAAATTGTTTAC  R : GAAAAGGTACTCAACCAAATA | 639 bp | 50 ^o^C |
| *erm* (C) | F : GCTAATATTGTTTAAATCGTCAAT  R : TCAAAACATAATATAGATAAA | 642 bp | 43 ^o^C |
| *erm* (F) | F : CGGGTCAGCACTTTACTATTG  R : GGACCTACCTCATAGACAAG | 466 bp | 50 ^o^C |
| *IntI1* | F : ATCATCGTCGTAGAGACGTCGG  R : GTCAAGGTTCTGGACCAGTTGC | 892 bp | 55 ^o^C |
| *Cassette* | F : GGCATCCAAGCAGCAAG  R : AAGCAGACTTGACCTGA | 750 ~  1907 bp | 57 ^o^C |

**Table S2. The Etest of *Campylobacter species* isolates from China Medical University Hospital stored bank.**

| **Antibiotics** | **Resistance** | **Breakpoints (μg/mL)** |
| --- | --- | --- |
| Amikacin | 5 (36%) | 4 (*E.coli*) |
| Ciprofloxacin | 8 (57%) | 4 |
| Imipenem | 2 (14%) | 0.25 (*E.coli*) |
| Tetracycline | 8 (57%) | 16 |

**Table S3. The *tet* genes distribution of plasmid DNA from *Campylobacter species.***

| **Strains (No.)**  ***tet* genes** | ***C. jejuni***  **(9)** | ***C. coli* (4)** | ***C. fetus* (1)** | **The number of positive reaction (%)** |
| --- | --- | --- | --- | --- |
| *tet* (A) | 8 (89%) | 3 (75%) | 0 (0%) | 11 (79%) |
| *tet* (B) | 0 (0%) | 0 (0%) | 0 (0%) | 0 (0%) |
| *tet* (K) | 0 (0%) | 0 (0%) | 0 (0%) | 0 (0%) |
| *tet* (L) | 2 (22%) | 1 (25%) | 0 (0%) | 3 (21%) |
| *tet* (M) | 7 (78%) | 3 (75%) | 0 (0%) | 10 (71%) |
| *tet* (O) | 8 (89%) | 2 (50%) | 1 (100%) | 11 (79%) |
| *tet* (Q) | 3 (33%) | 2 (50%) | 0 (0%) | 5 (36%) |
| *tet* (S) | 0 (0%) | 0 (0%) | 0 (0%) | 0 (0%) |
| *tet* (W) | 0 (0%) | 0 (0%) | 0 (0%) | 0 (0%) |

**Table S4. The *erm* genes distribution of plasmid DNA from *Campylobacter species.***

| **Strains (No.)**  ***erm* genes** | ***C. jejuni***  **(9)** | ***C. coli* (4)** | ***C. fetus* (1)** | **The number of positive reaction (%)** |
| --- | --- | --- | --- | --- |
| *erm* (A) | 0 (0%) | 0 (0%) | 0 (0%) | 0 (0%) |
| *erm* (B) | 2 (22%) | 1 (25%) | 0 (0%) | 3 (21%) |
| *erm* (C) | 0 (0%) | 0 (0%) | 0 (0%) | 0 (0%) |
| *erm* (F) | 0 (0%) | 0 (0%) | 0 (0%) | 0 (0%) |

**Table S5. Characteristics of various gene cassette types in class one integron of *Campylobacter species* isolates.**

| **Strains (No.)** | **Gene type** | **Gene size** | **Inserted gene cassette (5’- 3’)** | **Resistance pattern** |
| --- | --- | --- | --- | --- |
| *C. jeiunii* (9) | I | 1907 b.p. | *dfr12-gcuF-aadA2* | Trimethoprim, Streptomycin and Spectinomycin |
| *C. coli* (2) | I | 1907 b.p. | *dfr12-gcuF-aadA2* | Trimethoprim, Streptomycin and Spectinomycin |
| *C. coli* (1) | II | 750 b.p. | *dfrA7* | Trimethoprim |
